# Effectiveness and Safety of Combined Use of Home‐Based Radiofrequency Device and Arbutin Cream in Melasma and Facial Rejuvenation

**DOI:** 10.1111/jocd.70007

**Published:** 2025-02-07

**Authors:** Lei Zhang, Qian Zhao, Qilei Che, Wenju Wang, Tao Chen, Xiao Tang, Xin Zhao, Nianou Wang, Ke Li, Qingbiao Wa

**Affiliations:** ^1^ Medical Cosmetic Center Chengdu Second People's Hospital Chengdu China; ^2^ Department of Dermatology Chengdu Second People's Hospital Chengdu China; ^3^ Department of Ultrasonography Chengdu Second People's Hospital Chengdu China; ^4^ School of Biomedical Engineering Shanghai Jiao Tong University Shanghai China; ^5^ College of Pharmaceutical Sciences Zhejiang University Hangzhou China

**Keywords:** arbutin, facial rejuvenation, home‐based radiofrequency device, melasma, periorbital dermal thickness, skin hyperpigmentation, wrinkles

## Abstract

**Background:**

Melasma and facial aging are prevalent dermatological concerns. The combined use of a home‐based radiofrequency (RF) device and a topical depigmentation agent, such as an arbutin‐based cream, has shown potential in addressing both conditions.

**Aims:**

This study evaluated the effectiveness and safety of combining a home‐based RF device with arbutin cream to treat melasma and facial aging.

**Methods:**

This single‐center, open‐label study included 38 participants. The treatment involved using a home‐based RF device alongside an arbutin cream three times a week for 4 weeks, followed by a 4‐week rest period. Key outcomes included changes in modified Melasma Area and Severity Index (mMASI) scores, melanin index (MI), erythema index (EI), facial wrinkles, skin texture, and periorbital dermal thickness. The participants also provided a self‐assessment of the treatment efficacy.

**Results:**

After 4 and 8 weeks of treatment, compared with baseline, the mMASI scores decreased by 20.8% and 25.6%, whereas the MI decreased by 22.0% and 22.8%, respectively. The EI decreased by 5.94% and 6.86%, facial wrinkles decreased by 30.9% and 35.9%, and skin texture scores decreased by 19.7% and 21.4% at 4 and 8 weeks posttreatment, respectively. Significant improvements in periorbital dermal thickness and subcutaneous tissue thickness were observed after combined RF and arbutin cream treatment. None of the 38 participants experienced adverse events, and all reported satisfaction with the results.

**Conclusion:**

The combination of a noninvasive home‐based RF device and arbutin cream is effective in improving melasma and achieving facial rejuvenation, offering a safe and patient‐friendly treatment option.

## 1 Introduction

Melasma and facial aging are prevalent dermatological concerns. Melasma is a chronic, recurrent hyperpigmentation disorder caused by hyperfunctional melanocytes that deposit excessive amounts of melanin in the epidermis and dermis [1]. Facial aging is characterized by wrinkles, folds, reduced skin thickness, poor skin texture, and hyperpigmentation, including melasma [2, 3]. Developing effective treatments for melasma and strategies to mitigate facial aging remains a significant focus in medical aesthetics research.

The management of melasma is often challenging, with incomplete response in many cases and frequent relapses. A multimodality approach is required in most cases, incorporating photoprotection, topical depigmenting treatments, oral therapy, chemical peels, and laser and light therapy, based on the patient's characteristics and clinical presentation [4]. Although these approaches can lead to improvement, they are often associated with adverse effects such as skin redness, pain, and allergic reactions. Topical depigmentation agents, including hydroquinone and triple combination creams (fluocinolone–hydroquinone–tretinoin), remain the first‐line therapies for melasma [4], but their potential side effects restrict long‐term use. Similarly, antiaging treatments such as fillers, botulinum toxin, and minimally invasive surgical procedures are effective but involve higher risks. Consequently, there is a pressing need for safer and more effective solutions to address both melasma and facial aging.

Arbutin, a skin‐lightening agent derived from the bark and leaves of various plants such as bearberry, is a β‐d‐glucopyranoside derivative. It inhibits tyrosinase activity in melanocytes in a dose‐dependent manner, without compromising cell viability, demonstrating greater potency than kojic or l‐ascorbic acid [5]. Studies have shown that arbutin significantly reduces melanin production in humans [6, 7]. Additionally, it decreases reactive oxygen species by scavenging free radicals and enhancing antioxidant capacity [8]. Its antioxidant properties help to prevent cellular senescence and apoptosis while restoring mitochondrial function [9]. Despite its widespread use in cosmetics because of its safety profile, arbutin's efficacy is limited by poor skin penetration [10].

With the growing demand for convenient antiaging treatments, home‐based radiofrequency (RF) devices are gaining popularity, supported by research on their efficacy and safety. Studies demonstrate that RF is noninvasive, long‐lasting, safe, and effective across various skin types, making it ideal for home use [11, 12]. Moreover, RF technology has applications in drug delivery systems, facilitating the administration of active ingredients during dermatological treatments [13], similar to methods such as microneedling [14], electroporation [15], ultrasound [16], and iontophoresis [17]. RF enhances biofilm permeability by creating transient micrometer‐sized aqueous channels in the skin [18]. A prospective study involving 50 patients demonstrated that unipolar RF combined with transdermal delivery significantly improved Melasma Area and Severity Index (MASI) scores and reduced inflammatory responses [19]. Additionally, research by Ai et al. [20] indicates that a home‐based RF device effectively enhances skin texture and elasticity through a safe and well‐tolerated treatment protocol.

To address this, we investigated whether the combined use of RF and arbutin could effectively treat melasma and facial aging. This study aimed to evaluate the effectiveness and safety of a home‐based RF device in conjunction with arbutin cream for managing these conditions.

## 2 Materials and Methods

### 2.1 Study Participants

The cohort consisted of 38 participants with stable‐phase melasma and concurrent skin laxity and wrinkles.

The eligibility criteria included the absence of any other dermatological conditions and the nonuse of antiallergic medications within the preceding 3 months. Furthermore, all participants were mandated to use effective contraceptive measures throughout the study period. Prior to enrollment, each participant provided informed consent and confirmed their understanding of the study's scope and the procedures of the study.

Exclusion criteria included pregnancy, history of cardiac pacemaker insertion, active facial skin infections, metal implants, impaired sensation, tumors, ongoing retinoid or hormonal therapy, participation in other clinical trials, and recent phototherapy or injectable treatments within the past 12 months.

### 2.2 Treatment Protocol

The trial utilized the red‐light triangular RF instrument ARF001 (AMIRO, China) and a 7% Arbutin Cream (Obagi Medical Blend Fx Skin Brightening Cream, USA). Participants began each session by cleansing their faces, applying the arbutin cream, and activating the RF device. They selected the firm‐and‐lift mode and glided the device over their face for 5 min. This was then followed by a 15‐min fading and repair phase. To maintain hydration, the cream was reapplied promptly after the procedure. Treatments were conducted every other day for 4 weeks, followed by a 4‐week follow‐up period. In order to avoid the effects of ultraviolet (UV) radiation on melasma, sunscreens (Mentholatum Sunplay Skin Aqua UV Super Moisture Gel SPF50+, USA) were used throughout the process.

### 2.3 Study Design

This single‐center, open‐label, intraindividual controlled trial was designed to evaluate the effects of the red‐light triangular RF instrument and the arbutin cream on facial skin appearance. The initial 4 weeks encompassed thrice‐weekly utilization of both the RF device and arbutin cream. This was followed by a subsequent 4‐week period of discontinuation of the use of the RF device and arbutin cream. For the duration of the study period, participants agreed to use daily sunscreens. The treatment phase lasted for 4 weeks, with participants attending follow‐up visits conducted at baseline (0w), Week 4 (4w), and Week 8 (8w). Clinical grading and efficacy evaluations were performed by two independent physicians to ensure accuracy and consistency.

### 2.4 Test Environment

Strict environmental controls were implemented to ensure consistent and reproducible experimental conditions. The laboratory was shielded from direct sunlight, with a constant temperature of 22°C and a relative humidity maintained between 50% and 60%. Before each testing session (baseline, Week 4, and Week 8), the participants underwent standardized facial cleansing to remove sweat and cosmetic residues, to minimize external influences on the results. They then rested in a designated area for 30 min to stabilize their skin conditions. Comprehensive photographic assessments were conducted to document and analyze skin changes.

### 2.5 Skin Efficacy Evaluation Tests

Melasma severity was assessed using the modified Melasma Area and Severity Index (mMASI). Digital facial photographs were captured from the left, frontal, and right views under controlled environmental conditions to calculate the mMASI scores, which ranged from 0 to 24. Severity levels were categorized as mild (< 5), moderate (5–10), or severe (> 10), with higher scores indicating greater melasma severity [21].

Furthermore, the melanin index (MI) and erythema index (EI) were measured using a Mexameter (MX18; Courage & Khazaka, Koln, Germany) to evaluate changes in facial skin pigmentation throughout the treatment period.

Additionally, the VISIA‐CR photographic system (Canfield Imaging, NJ, USA), along with its analytical software, was used to measure critical parameters such as overall facial wrinkle density and skin texture. Stress Echo ultrasound (APLIO i800 TUS‐AI800; Canon Medical Systems, China) was used to assess changes in periorbital skin thickness before and after treatment. Dermal layer thickness was assessed at two key points: 0.5 and 1.0 cm lateral to the outer orbital rim.

### 2.6 Self‐Assessment

Data were collected 4 weeks after the intervention was concluded and 8 weeks posttreatment.

### 2.7 Statistical Methods

Data processing and analyses were performed using SPSS Statistics for Windows (version 19.0; IBM Corp, Armonk, NY). Repeated measures ANOVA was used to compare the two follow‐up periods. If Mauchly's test of sphericity was satisfied, the Greenhouse–Geisser result from the Tests of Within‐Subjects Effects was applied. Otherwise, the Pillai Trace results from multivariate tests were used. The mean values and standard deviations describe the quantitative variables reflecting the central tendency and variability. A significance threshold of *p* < 0.05 was established to ensure the robustness and validity of the analytical conclusions.

## 3 Results

### 3.1 Study Participants

This study included 38 female participants aged 28–60 years, with a mean age of 44 years. All participants completed the study without experiencing skin desquamation. Skin types ranged from Fitzpatrick type II to IV. No adverse events were reported during the study period.

### 3.2 Decrease in mMASI Scores

In this study, mMASI was used as the primary measure to evaluate melasma severity. As shown in Table 1, compared with baseline, the mMASI score decreased by 20.8% at 4w and 25.6% at 8w. Statistically significant differences were observed across all three time points (0w, 4w, and 8w). These findings indicate that the combination of a home‐based RF device and arbutin cream effectively reduced melasma within the initial 4‐week treatment period.

**TABLE 1:** Analysis of changes in mMASI scores before and after treatment.

	Baseline M ± SD	Week 4 M ± SD	Week 8 M ± SD	*F*	*p*
mMASI score	4.53 ± 1.33	3.59 ± 0.90	3.37 ± 0.60	32.10	**< 0.001**

*Note:* *p*‐Values < 0.05 are highlighted in bold.

### 3.3 Reduction in Melanin and Erythema

**FIGURE 1:**
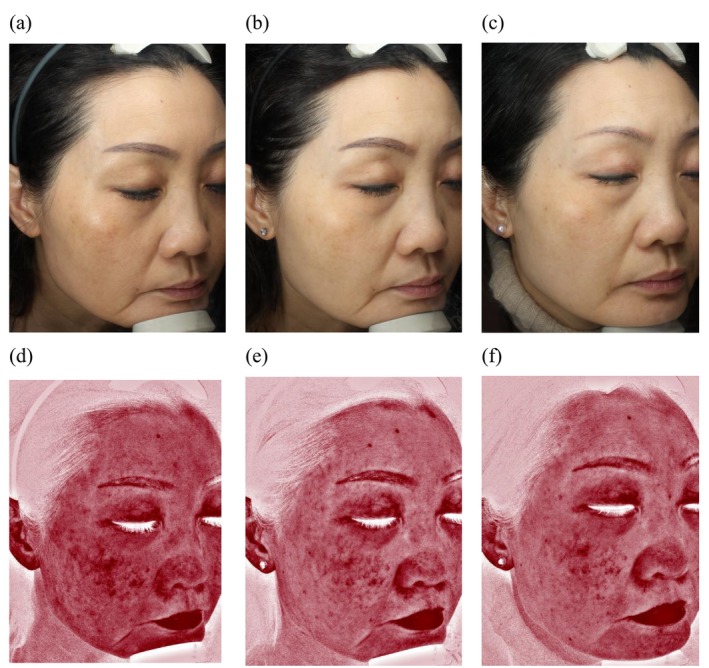
Graphical representation of melasma progression, telangiectasia, and erythema progression pre‐ and posttreatment. (a–c) Facial images for melasma progression captured using the VISIA system with white light at baseline (Week 0), at Week 4, and at Week 8; (d–f) Facial red area images for telangiectasia and erythema processed by RBX technology at baseline (Week 0), at Week 4, and at Week 8.

MI and EI levels in the anterior and posterior cheek skin were key assessment parameters in this study. As shown in Table 2, compared with baseline, MI decreased by 22.0% at 4w and 22.8% at 8w, whereas EI decreased by 5.94% at 4w and 6.86% at 8w. Statistically significant differences in both MI and EI were observed across all three time points (0w, 4w, and 8w). Additionally, the color intensity of melasma and erythema was significantly reduced at Weeks 4 and 8 compared with baseline, as illustrated in Figure 1a–c,e–g. These findings indicate that the combined use of a home‐based RF device and arbutin cream effectively improved melasma and reduced erythema.

**TABLE 2:** Results of the analysis of changes in melanin and erythema of the cheek skin before and after treatment.

	Baseline M ± SD	Week 4 M ± SD	Week 8 M ± SD	*F*	*p*
Melanin	196.73 ± 31.51	153.54 ± 31.65	151.79 ± 27.85	44.70	**< 0.001**
Erythema	304.49 ± 42.67	286.41 ± 48.74	283.59 ± 45.32	5.06	**0.01**

*Note:* *p*‐Values < 0.05 are highlighted in bold.

### 3.4 Improvement in Facial Aging

Using the VISIA photographic instrument, a comprehensive facial analysis focusing on wrinkles and skin texture was performed. As detailed in Table 3, compared with the baseline, facial wrinkles decreased by 30.9% at 4w and 35.9% at 8w, and texture scores decreased by 19.7% at 4w and 21.4% at 8w. There were statistically significant differences in wrinkles and texture at all three time points. These results underscore the efficacy of the combined application of the RF device and arbutin cream for wrinkle reduction and antiaging effects. These changes were sustained for at least 4 weeks posttreatment.

**TABLE 3:** Analysis of changes in VISIA facial skin indexes before and after treatment.

	Baseline M ± SD	Week 4 M ± SD	Week 8 M ± SD	*F*	*p*
Facial wrinkles	12.80 ± 8.29	8.84 ± 6.44	8.20 ± 6.36	15.21	**< 0.001**
Facial texture	3.04 ± 2.21	2.44 ± 1.57	2.39 ± 1.40	3.56	**0.039**

*Note:* *p*‐Values < 0.05 are highlighted in bold.

For ultrasound technology to assess periorbital skin thickness, we focused on dermal layer thickness at two key points: 0.5 and 1.0 cm lateral to the outer orbital rim. As detailed in Table 4, compared with baseline, periorbital dermal thickness (0.5 cm) improved by 19.0% (4w) and 19.0% (8w); periorbital dermal thickness (1.0 cm) improved by 26.8% (4w) and 31.7% (8w); subcutaneous tissue thickness (0.5 cm) decreased by −5.6% (4w) and 25.7% (8w); subcutaneous tissue thickness (1.0 cm) decreased by −1.9% (4w) and 27.0% (8w). There were statistically significant differences in dermal layer thickness and subcutaneous tissue thickness at the three time points. Therefore, these findings suggest that the combined application of a home‐based RF device and arbutin cream can stimulate collagen production in the dermal layer, enhance dermal thickness, and concurrently exert a tightening effect on subcutaneous fat.

**TABLE 4:** Analysis of changes in skin thickness of the outer corners of the eyes before and after treatment.

Measurement sites	Baseline M ± SD	Week 4 M ± SD	Week 8 M ± SD	*F*	*p*
Dermal layer thickness (0.5 cm)	0.42 ± 0.10	0.50 ± 0.25	0.50 ± 0.09	7.66	**0.002**
Dermal layer thickness (1.0 cm)	0.41 ± 0.10	0.52 ± 0.21	0.54 ± 0.14	8.31	**0.001**
Subcutaneous tissue thickness (0.5 cm)	1.44 ± 0.36	1.52 ± 0.37	1.07 ± 0.56	14.25	**< 0.001**
Subcutaneous tissue thickness (1.0 cm)	1.59 ± 0.42	1.62 ± 0.36	1.16 ± 0.58	12.92	**< 0.001**

*Note:* *p*‐Values < 0.05 are highlighted in bold.

### 3.5 Subject Self‐Assessment: Satisfaction Evaluation

**FIGURE 2:**
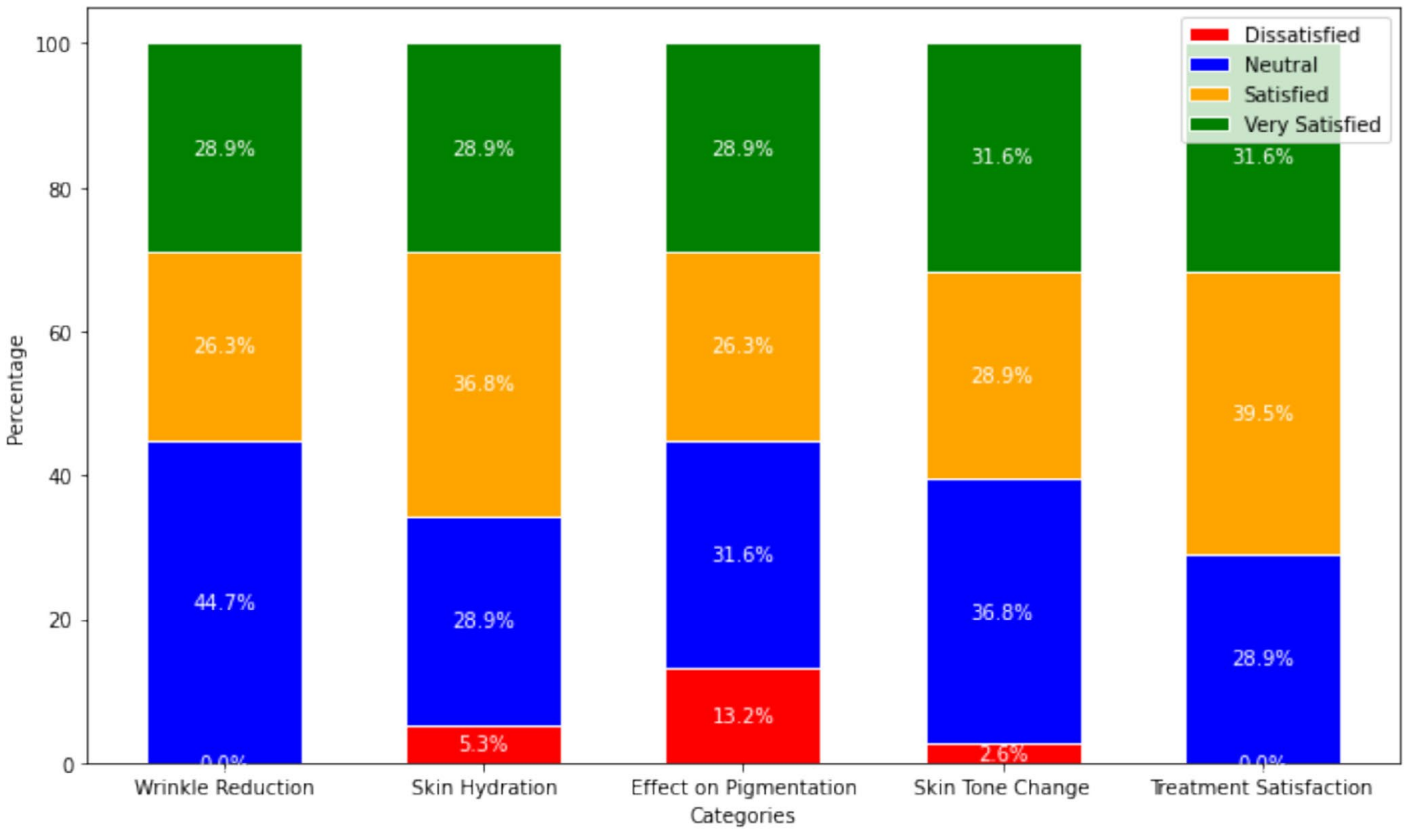
Graph analyzing subjects' self‐assessment results.

Upon completing the treatment, the participants evaluated their satisfaction with the outcomes using a questionnaire. This evaluation aimed to capture the subjective effects of the treatment from the patient's perspective, providing valuable feedback on the intervention's perceived effectiveness. As illustrated in Figure 2, over 55% of participants reported being “satisfied” or “very satisfied” with improvements in wrinkle reduction, skin hydration, pigmentation effects, skin tone changes, and overall treatment outcomes. Dissatisfaction rates for skin hydration, pigmentation effects, and changes in skin tone were 5.3%, 13.2%, and 2.6%, respectively. Notably, no instances of dissatisfaction were reported for wrinkle reduction or overall dissatisfaction with treatment outcomes. Overall, participants expressed high levels of satisfaction with the combined use of the home‐based RF device and arbutin cream.

## 4 Discussion

In recent years, as patients are increasingly seeking effective treatments with minimal recovery time and fewer side effects, noninvasive cosmetic procedures have gained significant popularity in dermatology. Combining topical agents with noninvasive technologies, such as RF, has been shown to enhance treatment outcomes for conditions like melasma [22, 23]. RF devices have been shown to promote collagen remodeling and improve skin texture by delivering controlled heat to the dermal layers [22, 23]. Consistent with these findings, our research demonstrated that combining the RF device with arbutin cream not only reduced hyperpigmentation but also improved the overall skin appearance. The depigmenting effect of arbutin, mediated through tyrosinase inhibition, complements RF's collagen‐stimulating properties, offering a comprehensive approach to skin rejuvenation.

Furthermore, the observed reduction in erythema during the treatment period suggests that this combined approach is both effective and safe for individuals with sensitive skin types. This finding is particularly significant, as traditional melasma treatments, such as chemical peels and laser therapy, are often associated with a risk of postinflammatory hyperpigmentation, especially in individuals with darker skin tones.

However, the absence of control groups using either the RF device or arbutin cream independently limits our ability to definitively conclude that the combined therapy is superior. In this study, after 4 and 8 weeks of combined treatment, the mMASI scores decreased by 20.8% and 25.6%, respectively, and the MI decreased by 22.0% and 22.8%, respectively. In comparison, a study by Kwon et al. where RF was used alone reported MI reductions of 10.4%, 11.9%, and 13.7% at 3, 6, and 9 weeks, respectively [24]. This suggests that the combination of RF and arbutin was more effective than RF alone. Morag et al. studied the use of arbutin cream alone, where MI decreased by 4.9% and 8.9%, respectively [7]. Similarly, Zhang et al. studied the use of arbutin cream alone, where mMASI decreased by 6.8% and 12.6%, and MI decreased by 2.8% and 5.3%, respectively, compared with baseline [25]. These comparisons underscore the added benefit of combining the RF device with arbutin cream for the treatment of melasma.

Despite these promising results, this study had several limitations. The relatively small sample size and the 8‐week observational period may have influenced the robustness of the findings. However, the combined treatment approach in this study is notable for its uniqueness, convenience, and safety compared with previous studies. Future research should aim to increase the sample size, extend the treatment and observational periods, and incorporate direct comparisons with other standard therapies. Additionally, mechanistic studies exploring the combined effects of RF treatment and arbutin cream on melasma and achieving facial rejuvenation are warranted to provide stronger evidence and expand clinical options.

In conclusion, the combined use of a noninvasive, home‐based RF device and arbutin cream effectively improved melasma and facilitated facial rejuvenation, demonstrating a safe and well‐tolerated treatment protocol.

## Conflicts of Interest

The authors declare no conflicts of interest.

## Data Availability

The data that support the findings of this study are available from the corresponding author upon reasonable request.
